# Regeneration in *Stentor coeruleus*

**DOI:** 10.3389/fcell.2021.753625

**Published:** 2021-09-29

**Authors:** Wallace F. Marshall

**Affiliations:** ^1^Department Biochemistry and Biophysics, University of California, San Francisco, San Francisco, CA, United States; ^2^Chan Zuckerberg Biohub, San Francisco, CA, United States

**Keywords:** ciliates, cellular regeneration, cellular wound healing, evolution of metazoan, morphogenesis

## Abstract

We often think about regeneration in terms of replacing missing structures, such as organs or tissues, with new structures generated via cell proliferation and differentiation. But at a smaller scale, single cells, themselves, are capable of regenerating when part of the cell has been removed. A classic model organism that facilitates the study of cellular regeneration in the giant ciliate *Stentor coeruleus*. These cells, which can grow to more than a millimeter in size, have the ability to survive after extensive wounding of their surface, and are able to regenerate missing structures. Even a small piece of a cell can regenerate a whole cell with normal geometry, in a matter of hours. Such regeneration requires cells to be able to trigger organelle biogenesis in response to loss of structures. But subcellular regeneration also relies on intracellular mechanisms to create and maintain global patterning within the cell. These mechanisms are not understood, but at a conceptual level they involve processes that resemble those seen in animal development and regeneration. Here we discuss single-celled regeneration in *Stentor* from the viewpoint of standard regeneration paradigms in animals. For example, there is evidence that regeneration of the oral apparatus in *Stentor* follows a sender-receiver model similar to crustacean eyestalk regeneration. By drawing these analogies, we find that many of the concepts already known from the study of animal-scale regeneration and development can be applied to the study of regeneration at the cellular level, such as the concepts of determination, induction, mosaic vs. regulative development, and epimorphosis vs. morphallaxis. We propose that the similarities may go beyond analogy, and that some aspects of animal development and regeneration may have evolved by exploiting pre-existing subcellular developmental strategies from unicellular ancestors.

## Introduction

The ability to heal wounds and regenerate is a fundamental feature that separates living from non-living systems. Regeneration, which we view as the ability of a living thing to re-build missing parts following their accidental or deliberate removal, has long been the subject of intense investigation, partly because it is a fascinating process in its own right, but even more so because it sheds light on the process of development.

Given the clear importance of stem cells such as neoblasts in regenerating tissues and organs in animals, studies of regeneration have justifiably focused on the mechanisms for replacing dead or lost cells with new cells that have taken on the appropriate differentiation state (Tanaka and Reddien, 2011). However, it turns out that even within individual cells, missing parts can regenerate. In many cells types, including both free-living organisms and cells inside the human body, cilia can be regenerated following their loss from mechanical shearing or other forms of stress (Rosenbaum and Child, 1967; Ibrahim et al., 1979; Heller and Gordon, 1986; Atef et al., 2009). Neurons are capable of regrowing dendrites or axons that have been damaged or removed (Baas and Heidemann, 1986; Hall and Cohen, 1988; Maier and Schwab, 2006; Bloom and Morgan, 2011), and hair cells of the ear are capable of regenerating stereocilia following their shearing by loud noises (Cotanche, 1987). A classic example of cellular regeneration is the ability of the giant green alga Acetabularia to regenerate its cap structure (Mine et al., 2008).

The examples just cited all represent cellular protrusions of various forms, which are prone to shearing and therefore in particular need of regenerative mechanisms. Whether or not internal organelles can regenerate is a question that calls for more investigation. One case where this has been studied is the Golgi complex, which can be induced to resorb via treatment with brefeldin. When the drug is washed out and normal membrane trafficking is restored, the Golgi is re-built inside the cell (Langhans et al., 2007; Ito et al., 2012). When organelle inheritance to the bud is blocked in budding yeast, the daughter cells are often still able to re-form the organelle via independent biogenesis mechanism that do not require inheritance of the pre-existing organelle from the parent cell (Jin and Weisman, 2015).

Going beyond specific structures, some cells are able to regenerate completely from tiny fragments, which requires not only the re-building of lost or damaged structures, but also the re-arrangement of cellular components to restore a normal cell architecture. Regeneration of cells from cell fragments has been most extensively studied in large single-celled protists, mainly amoeba and ciliates, whose large size makes the surgery easy (Balamuth, 1940). Several examples of cells which have been shown capable of restoring a normal size and shape after being cut into pieces include the giant ciliates *Stentor* (Tartar, 1961) and *Blepharisma* (Kumazawa, 1979) as well as giant Amoebas (Radir, 1931; Goetz Von Olenhusen et al., 1979). Just as the study of regeneration has shed light on the mechanisms of animal development, studies of regeneration at the subcellular level have the potential to reveal the mechanisms that determine the geometry of cells.

Can all cells regenerate? One of the confusing aspects of animal regeneration is the extent to which different species, and even whole phyla, differ in their regenerative capacity. Some species, such as hydra or flatworms, can regenerate entire organisms from tiny fragments, while in other cases, regeneration is restricted to smaller portions such as limbs or fingertips. One of the goals of studying regeneration has always been to see if there is a way to increase the ability of humans to regenerate following injury or degeneration, with spinal cord neurons being a system of particular interest.

The same variability in regenerative capacity seen across animals is also seen among single cells. One obvious difference among cell types is the number of nuclei. Only a cell fragment that contains the nucleus will be able to regenerate and continue living. This is of course self-evident in light of modern understanding of genomes, but prior to that understanding, it was directly demonstrated that regeneration in both amoeba and ciliates depends on the presence of a nucleus in the regenerating fragment, and could be restored to enucleated fragments by nuclear transplantation (Tartar, 1961). But even among phyla with similar sizes and distributions of nuclei, there are differences in regenerative potential. A general trend in the literature is that the cells that can regenerate from the most dramatic fragmentation and surgery tend to be very large cells, such as *Xenopus* oocytes, giant amoebas or the giant ciliates *Stentor* and *Blepharisma* (Tartar, 1961; Sonnemann and Bement, 2011). Large size might itself be important by allowing cells to survive longer after wounding. The larger the volume of the cell, the more time it would take to “bleed out” in the sense of losing cytoplasm to the medium or undergoing damaging changes in cellular chemistry. But there are also differences in regenerative ability seen even when differences in wound healing are not at play. For example, when the large ciliate *Stentor* is bisected, the two fragment cells each restore a completely normal shape. In contrast, when a different ciliate, *Paramecium*, is bisected, the partial cells often fail entirely to regenerate (Calkins, 1911) and when they do, they tend to maintain whatever positioning of structures were present prior to the cut, so that they do not restore a normal cell geometry (Tartar, 1954a). Similarly, re-arrangements of the rows of cilia on the cortex of a *Paramecium* cell can persist indefinitely, suggesting that in this species cells either cannot detect, or cannot repair, geometrical rearrangements of cellular organization (Beisson and Sonneborn, 1965). The differing capacities of different cell types to restore proper global organization following cutting or perturbation is directly equivalent to the difference between mosaic and regulative development in animal embryos. From this viewpoint, we would say that *Stentor* development is regulative while *Paramecium* development is mosaic.

Eggs after fertilization represent an interesting gray zone between single and multicellular life. In phyla with mosaic development, much of the body plan is already determined by regional differences inside the egg prior the first cleavage division. Can this patterning be regenerated when the embryo is still just a single cell? Depending on the species, when embryos are dissociated into blastomeres at early cleavage divisions, sometimes the individual blastomeres can regenerate whole organism, such as in the case of sea urchin embryos (Driesch, 1891). In other cases, such as the limpet *Patella*, isolated blastomeres will give rise to precisely those tissues that they normally would give rise to, but cannot regenerate any other parts of the animal, indicating that developmental fate may have already been specified (Wilson, 1904). Such specification of fate at such an early stage clearly indicates that the egg has been regionalized prior to cleavage. Indeed, it can be directly seen in some species that fate-determining mRNA molecules have a polarized distribution within the egg before the first cleavage division (Nishida, 2005; Sardet et al., 2007). Such spatial segregation of fate determinants requires mechanisms to partition these fate determinants in distinct parts of the egg, in other words, a mechanism to establish spatial variation or geometry within a cell. Since this is the same type of problem that unicellular organisms need to solve when they divide and regenerate, it may well be the case that multicellular organisms have co-opted pre-existing mechanisms for regeneration and development of pattern in single-celled ancestors. Testing this hypothesis will require a mechanistic understanding of regeneration in unicellular organisms. Even if it turns out that regeneration in protists is completely different from regeneration in animals, we believe that an attempt to compare the two may still shed light on both types of regeneration. Unicellular organisms have a number of advantages for studying pattern formation and development at the subcellular level. Their large size makes microsurgery possible at a level that would be extraordinarily difficult in, say, mammalian cells. They are naturally free-living such that studying individual cells in the lab is possible, without the usual concerns that exist with cultured animal cells grown outside of their normal 3D tissue context. Because they are free-living, there is less concern about the possibility that subcellular patterning is driven by cues provided by neighboring cells in a tissue, such that attention can be focused on intracellular patterning mechanisms. Finally, many unicellular protists have elaborate surface structures that allow patterning to be easily visualized in living cells (Aufderheide et al., 1980), in much the same way that bristle patterns were used to visualize patterning in *Drosophila* embryos in the original Heidelberg screens for patterning mutants (Nüsslein-Volhard and Wieschaus, 1980).

**FIGURE 1:**
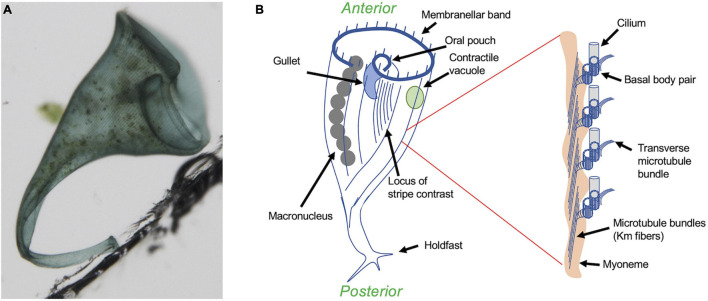
Overview of *Stentor coeruleus*. **(A)** Micrograph of a single *Stentor* cell, attached to the wall of a plastic chamber via its holdfast. **(B)** Anatomy of *Stentor*. The oral apparatus (OA) is located at the anterior end of the cell and consists of a membranellar band of cilia, an oral pouch where food is temporarily captured, and a gullet where food is ingested. Inset shows expanded view of the region circled in red, illustrating the ultrastructural organization of the ciliary rows. Each row contains not only pairs of basal bodies, one of which nucleates a cilium, but also a parallel array of microtubule bundles known as Km fibers (Huang and Pitelka, 1973) and, underneath the microtubules, a contractile fiber bundle known as a myoneme, which is composed of centrin-like EF hand calcium binding proteins (Maloney et al., 2005). An additional set of microtubule bundles, known as transverse microtubules, emerge from each basal body pair and extend perpendicularly to the Km fibers toward the adjacent ciliary rows. The spaces in between these rows are filled with blue pigment, giving rise to the blue color seen in **(A)**. The spacing between the ciliary rows shows a circumferential variation, such that the spacing between the rows, and hence the width of the intervening blue stripes as well as the lengths of the transverse microtubule bundles, starts out large at one side of the cell and then gradually decreases as one moves around the circumference, until eventually the narrowest stripes (mostly closely spaced ciliary rows) about the widest stripes (mostly widely separated ciliary rows). This region is known as the locus of stripe contrast, and represents a key site for regeneration of oral structures.

Among the protists, ciliates have a highly visible surface patterning that has made them particularly useful as model organisms for studying the mechanisms of pattern formation within cells (Aufderheide et al., 1980; Frankel, 1989). This review will focus on regeneration in the giant heterotrichous ciliate *Stentor coeruleus* (Figure 1A), arguably the best-studied model for single-cell regeneration due to its large size and prodigious powers of wound healing (Tang and Marshall, 2017; Zhang et al., 2021) that allow it to survive almost any cutting and grafting experiments that have been attempted. Another advantage of *Stentor* for studying regeneration is its dramatic blue body striping that provides a natural set of fiduciary marks to assess cellular pattern in living cells. These blue stripes (Figure 1A) reflect the organization of the *Stentor* cortex as a parallel array of ciliary rows (also known as “kineties”) which consists of rows of basal bodies with associated microtubule bundles (Figure 1B inset). A blue pigment (Stentorin) is present in the gaps between the ciliary rows and gives rise to the stripe like appearance of the cell surface. Overall, the *Stentor* cell shows a clear anterior-posterior polarity, with an oral apparatus (feeding organelle) at the anterior end, and a holdfast at the posterior end. The body striping is non-uniform in width—on one side of the cell, the ciliary rows are spaced relatively far apart from each other, such that the intervening blue stripes are wide. As one progresses around the cell, the ciliary rows become progressively closer together such that the blue stripes become narrower. Eventually a point is reached where the narrowest stripes meet the widest stripes, a region call the locus of stripe contrast or the contrast zone. This contrast zone conventionally defines the ventral side of the cell body, thus producing a dorsal-ventral axis perpendicular to the A/P axis. These two body axes define a midline, and it turns out that every visible cellular structure has a defined left-right position relative to this midline. For example, the macronucleus, which contains thousands of copies of the genome, is located to the right of the midline, while the contractile vacuole, an organelle that collects and expels excess water to maintain osmotic balance (Allen and Naitoh, 2002), is located to the left. The *Stentor* cell thus possesses the same body axes that a bilaterian animal does.

*Stentor* can regenerate following a vast range of surgical perturbations (Tartar, 1961), but despite over a century of experimental work on *Stentor* regeneration, we still know virtually nothing about how this cell regenerates at a molecular mechanistic level. Rather than attempt to exhaustively review the hundreds of surgical experiments reported in *Stentor*, we will focus on four specific regeneration paradigms: regeneration of the oral apparatus, regeneration of the posterior holdfast, regeneration following bisection into anterior and posterior halves, and finally recovery of body wall pattern following disarrangement of the cortex. Each of these regenerative paradigms gives us clues about how *Stentor* may detect abnormalities in its geometry as well as how those abnormalities are corrected, and together they will allow us to ask what similarities and differences can already be discerned between *Stentor* and better-known animal models for regeneration.

## Regeneration of the Oral Apparatus in *Stentor*

**FIGURE 2:**
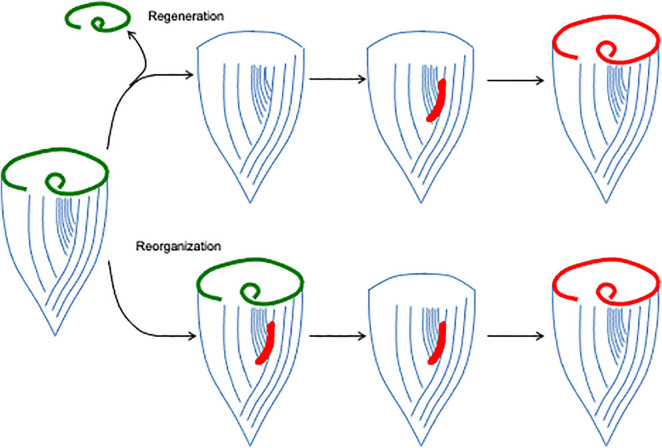
Oral apparatus regeneration and reorganization in *Stentor*. When the original oral apparatus (green) is removed, an oral primordium (red) forms at the locus of stripe contrast. This primordium, consisting of thousands of basal bodies, organizes into a new oral apparatus as it migrates to the anterior end of the cell. The same process can also occur spontaneously, creating a reorganization in which the old oral apparatus is replaced by a new, usually larger one.

The most intensively studied regenerative process in *Stentor* is regeneration of the oral apparatus (OA), a complex structure (Paulin and Bussey, 1971) consisting of a membranellar band surrounding a frontal field of cilia that together create a feeding flow to capture food, an oral pouch into which food is swept, and a gullet through which food is ultimately ingested via endocytosis (Figure 1B). The membranellar band itself is a large ring of “membranelles,” each of which consists of parallel rows of cilia that form and beat together as a group. The entire oral apparatus can be removed by surgery or by treatment with sucrose or other noxious chemicals that trigger an autotomy process in which all or part of the oral apparatus is shed (Tartar, 1957b). Once the oral apparatus is removed, a new one begins to form at the locus of stripe contrast on the ventral surface of the cell, where the narrow and wide surface stripes meet (Figure 2). Formation of a new oral apparatus proceeds through an intricate series of morphological steps, beginning with formation of thousands of basal bodies, which then arrange themselves into orderly rows and then sprout cilia to produce functional membranelles. Once the membranelles have formed, the cortex of the cell undergoes a rearrangement such that a patch of ciliary rows to the right of the membranellar band curl to form the frontal field, along with the membranellar band itself that curves into its final position. At the same time, the oral pouch and gullet develop at the posterior end of the membranellar band. The formation of the oral structures represents an instance of the embryological concept of determination but at a subcellular level, in that if the posterior end of the oral primordium is removed, no gullet will subsequently form (Tartar, 1957a). Formation of a new oral apparatus also happens spontaneously at apparently random times throughout the life of a cell, in a process known as “reorganization.” This process is thought to play a role in maintaining the usual scaling relation between the size of the oral apparatus and the size of the whole cell. *Stentor* cells double in size between divisions, such that the OA a cell is born with will eventually become too small. When the cell becomes disproportionately large compared to the current size of its OA, it will reorganize, shedding all or part of its old OA and replacing it with a newer, larger one. As in regeneration, the new OA in reorganization forms at the locus of stripe contrast and proceeds through the same set of morphological steps, indicating that it is the same morphological process. The same sequence of morphogenetic steps as seen in OA regeneration and re-organization is also seen during cell division, a topic we will discuss below in section “General Issues of Regeneration Shared Between *Stentor* and Animals.”

**FIGURE 3:**
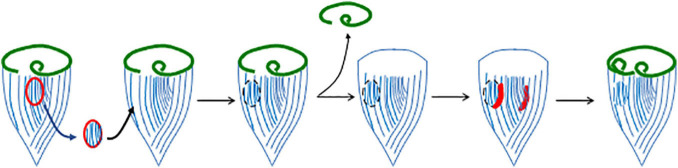
Induction of a new oral primordium by juxtaposition of narrow and wide striped cortical regions. In this experiment, a region of the cortex containing narrow stripes (closely spaced ciliary rows), but not including the locus of stripe contrast itself, is removed from one cell and grafted onto another in a region containing wider stripes. When the graft recipient cell is induced to regenerate, the new contrast zone supports the formation of a second oral primordium.

One of the most interesting features of OA regeneration is the role of the stripe contrast zone in this process. If the contrast zone is surgically removed and transplanted onto another cell, it will cause the recipient cell to form a second oral primordium during regeneration, thus acting much like an “organizer” in animal development (Tartar, 1956a). There is clearly something special about the locus of stripe contrast, since it always predicts the site where the new oral primordium will form, but what is the nature of the determinant? One possibility is that the contrast in stripe width is a consequence of some molecular mark at that site, such as a localized protein or mRNA, which also dictates oral primordium position. However, surgical experiments suggest that it is actually the contrast in stripe width itself, rather than some pre-existing mark at the contrast zone, that is important. If new contrast zones are created surgically, by grafting a patch of narrow stripes into a region of wide stripes on the back of the cell (Figure 3), a new oral apparatus will form at this ectopic contrast site, indicating that the contrast in stripe width is actually sufficient.

Regeneration of the oral apparatus requires the continuous presence of the nucleus. If the nucleus is removed during regeneration, the process grinds to a halt (Tartar, 1961), presumably reflecting a need for gene expression at multiple stages of regeneration. Studies with inhibitors of transcription and translation are consistent with this view (Whitson, 1965; James, 1967; Burchill, 1968; Younger et al., 1972), as is the fact that an increase in transcription is directly detectable during regeneration (Ellwood and Cowden, 1966; Burchill, 1968; Younger et al., 1972). It is interesting to consider how a sequential program of gene expression, acting as a “production schedule,” may contribute to the orderly events of oral apparatus development. The fact that oral regeneration requires gene expression has allowed RNA sequencing studies to investigate the process by asking which genes are turned on at each stage in the process (Sood et al., 2017; Onsbring et al., 2018; Wei et al., 2020). By inhibiting translation at the start of regeneration, it was possible to show that the regeneration program is organized as a cascade, such that a small number of early genes must be translated in order to trigger transcription of the later genes (Sood et al., 2021).

**FIGURE 4:**
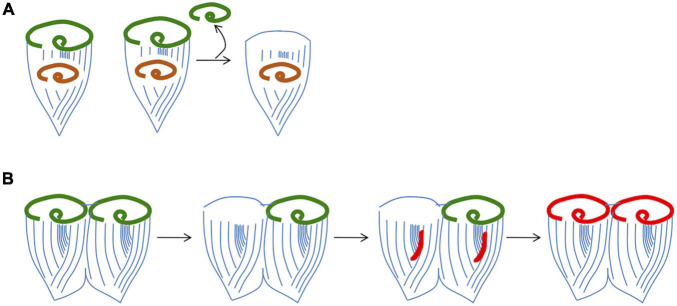
Evidence that regeneration is inhibited by a signal from the existing oral apparatus. **(A)** Implantation of OA material into a cell prevents regeneration. The implanted OA is denoted by orange color. **(B)** Removal of one OA from a grafted doublet cell is able to trigger parallel regeneration and reorganization.

One fundamental outstanding question is what cue triggers formation of a new oral apparatus during regeneration. One model is that the existing oral apparatus sends out an inhibitory signal, such that as long as it is present, the cell will not form a new oral primordium. Such a model was suggested by surgical experiments (Figure 4A) in which implantation of an additional oral apparatus is reported to block regeneration even when the original oral apparatus of a cell is removed (Hyvert et al., 1972). Grafting an oral apparatus back onto the anterior end of a regenerating *Stentor* causes regeneration to cease and the oral primordium to be resorbed (Tartar, 1958), suggesting that the inhibitory signal can act for a prolonged period, not just at the very first step of regeneration. The situation is apparently more complex than a simple diffusible signal, however, based on other experiments showing that displacement of the oral apparatus within the cell can trigger regeneration. In these experiments, the cortex is cut and the oral apparatus rotated or transplanted to other regions of the cell. Normally, the oral pouch and gullet are located anterior to the stripe contrast zone. Whenever this arrangement is perturbed, regeneration is triggered. For example, if a cut is made through the cortical rows and the anterior part of the cell is rotated relative to the rest of the cell, thus moving the oral pouch and gullet out of alignment with the contrast zone, this is sufficient to trigger formation of a new oral primordium (Tartar, 1956b). These observations are consistent with a sender-receiver model in which an inhibitory signal is generated at the oral apparatus and then transmitted along the cortical rows to the contrast zone, where it acts to suppress regeneration as long as the oral apparatus is present. The nature of this signal is currently unknown. De Terra further implicated the role of the cortical microtubules in transmitting an inhibitory signal by showing that when a ring of cortical rows is inserted in reverse orientation to the rest of the body wall cilia between the OA and the contrast zone, regeneration is triggered even though an intact OA is still present (de Terra, 1985). In doublet cells from which one OA is removed (Figure 4B), the corresponding oral primordium is activated to regenerate a replacement OA. At the same time, the oral primordium in the other half of the doublet cell is also activated, such that it undergoes a reorganization (Tartar, 1954b). Taken together these experiments indicate that a single missing OA is sufficient to activate the regeneration program as long as it is connected to a contrast zone by correctly oriented ciliary rows, but once it has triggered development of an oral primordium, some signal can spread to the rest of the cell and thereby activate other contrast zones that may still have an intact associated oral structure. The phenomenon of reorganization also raises questions about the regulation of oral primordium activation. During reorganization, a new OA is formed even in the presence of an existing one. Given that reorganization can be triggered by a mismatch in organelle size (e.g., when the OA is disproportionally small relative to the cell body), the phenomenon may indicate some presently unknown link to cell size. In fact, understanding reorganization may provide a way to learn about how cells size both organelle and cell size. Clearly, the regulatory logic of OA regeneration is not as simple as a diffusible beacon signal that directly triggers target genes. Unraveling the complexities of this regulatory system will require information about the molecules involved.

## Restoration of Global Anterior-Posterior Polarity in Regenerating *Stentor*

A normal *Stentor* cell has a clear anterior-posterior polarity (Figure 1B). This polarity includes not just the presence of the OA at the anterior end, but also a holdfast at the posterior, and it extends to virtually all components of the cell, each of which has a well-defined position along this axis. The ubiquitous cortical ciliary rows, with their associated microtubule Km fibers, align themselves parallel to the A/P axis. *Stentor* is able to regenerate structures at the posterior, just as it does the OA at the anterior, and can in fact do both at once when cells are cut into pieces. These regeneration processes, along with the striking ability of *Stentor* to recover a normal architecture when its entire cortex is randomly disarranged by minceration (Tartar, 1960), points to a regulative process for ensuring global cell organization in much the same way that an animal embryo has mechanism to ensure global organization of its body plan. We begin our discussion of pattern regulation by considering the posterior-most structure of the cell as the basis for further discussion of the A/P axis.

**FIGURE 5:**
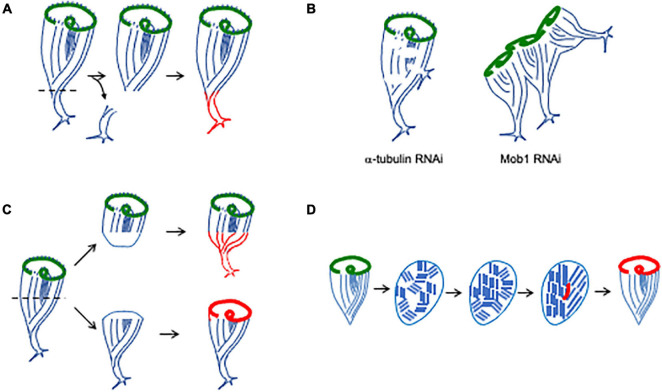
Regenerating and maintaining proper cell shape. **(A)** Regeneration of holdfast. Newly formed structures are indicated in red. **(B)** Identification of molecules involved in maintaining a single unified anterior-posterior axis (Slabodnick et al., 2014). Tubulin knockdown causes the ciliary rows to become discontinuous, and additional holdfasts begin to sprout from the sides of the cell body. Mob1 RNAi causes cells to form multiple holdfasts and a garland-like arrangement of oral apparatus, one for each posterior pole. **(C)** Regeneration of bisected cells. Anterior fragment inherits the pre-existing OA and regenerates a holdfast. Posterior fragment inherits the pre-existing holdfast and regenerates an OA. Note that the half-cells start out with abnormally short and squat shapes, and the anterior fragment has a disproportionately large OA. Properly proportioned shape and sizes of components is gradually established over a period of hours (Morgan, 1901). **(D)** Regeneration following cortical disarrangement. When cells are minced into pieces, the cortical rows break up into fragments. Over time, these fragments merge, either by growth or rotation, eventually restoring parallel rows. When a region of stripe contrast emerges, an oral primordium (red) forms, leading to formation of a new oral apparatus.

At the posterior end of the cell is a holdfast structure that the cell uses to attach to the substrate during filter feeding. Regeneration of the holdfast following its surgical removal (Figure 5A) is extremely rapid, taking place on the time scale of tens of minutes (Tartar, 1961). Unlike oral apparatus regeneration, holdfast regeneration does not require the nucleus. The molecular components of the holdfast are not known, hence there is little we can say about the molecular processes of holdfast assembly. Normally, the holdfast forms where the microtubule bundles on the cell surface terminate at their minus ends. This fact suggests a simple model for how the cell could know where to build the hold fast—by targeting molecules to the minus ends of the bundles, either via microtubule end-binding proteins or using motor proteins that move toward the minus ends. On the other hand, one could argue that there is some other factor that determines the posterior most region of the cell, and that this posterior determinant both triggers holdfast formation and also joins the minus ends of the microtubule bundles into a confined region.

Direct evidence that the minus ends are in fact sufficient to trigger holdfast assembly comes from experiments in which the cortical rows are surgically perturbed. When cuts are made or the cortex re-arranged such that a group of microtubule minus ends are ectopically created far from the posterior pole of the cell, a new holdfast immediately grows from this position (Tartar, 1961). The same effect is seen when tubulin is depleted using RNAi (Figure 5B). In this case, as tubulin protein is depleted, the cortical rows become less and less continuous, and “holes” start to appear in which bundles can be seen to terminate far from the posterior pole (Slabodnick et al., 2014). At the same time, ectopic holdfasts sprout from the side of the cell. These are not just morphologically similar to the holdfast; they can actually serve to anchor the cell (Slabodnick et al., 2014). These results are thus consistent with a model in which the holdfast forms wherever the microtubule bundles end. According to this model, when breaks in the cortex appear due to depletion of tubulin, this holdfast-inducing molecule erroneously accumulates at the minus ends of the bundles around the break, and cause the formation of an ectopic holdfast.

The position of the holdfast seems to be coordinated relative to that of the oral apparatus, presumably because the parallel microtubule bundles of the ciliary rows are anchored at the base of the OA and then run down to the other end of the cell. When extraneous posterior halves are grafted onto a cell, they gradually coalesce to form a single posterior pole on the exact opposite end of the cell from the OA (Tartar, 1961). One molecular candidate is now known that appears to play a role in this process. RNAi of the highly conserved kinase scaffolding protein Mob1 produce cells with a “medusoid” appearance, in which a garland of OAs at the anterior of the cell are matched with multiple posterior poles, with parallel microtubule bundles linking each OA in the garland to a corresponding posterior pole complete with a functional holdfast (Figure 5B; Slabodnick et al., 2014). These results suggest that Mob1 is part of the mechanism that normally ensures a single unified A/P body axis in the *Stentor* cell. Time-lapse imaging suggests that the first morphological defect in Mob1 RNAi cells is a failure to properly position a new OA during spontaneous reorganization. A new posterior pole then sprouts from the cell opposite to the location of the new OA, which suggests a long-range interaction of some sort whereby the OA dictates the location of the posterior pole, possibly by organizing ciliary rows into a coherent group that is perpendicular to the edge of the OA itself.

When cells are cut into pieces, it becomes necessary to re-build both anterior and posterior structures. When a *Stentor* cell is cut in half transversely the two halves, anterior and posterior, will each recover a normal cell form (Figure 5C). The anterior half-cell contains the oral apparatus of the original cell and therefore needs only to grow a new holdfast. The posterior half-cell contains the old holdfast but needs to form a new oral apparatus. Thus, regeneration after bisection ends up entailing the two processes already discussed—regeneration of oral apparatus and holdfast. Regeneration is possible in both half-cells because of the elongated shape of the macronucleus. When a cell is bisected, each half retains a portion of the macronucleus, which is highly polyploid (most genes are present a copy number of approximately 50,000 copies per cell; Slabodnick et al., 2014). Thus, each half cell retains many copies of the genome. However, there is a complication caused by the fact that the two half cells are only half the size of the starting cell. It is a general phenomenon in most cells that the size of their organelles and other structures scales with the size of the whole cell, and this is also true for *Stentor*. Larger cells have larger oral apparatuses, and cells maintain a relatively constant ratio of length to diameter as they grow (Morgan, 1901). When a cell is bisected, the two halves are abnormally short given their width, and the oral apparatus in the anterior half is twice as large as would be appropriate for a small cell of that size. Thomas Hunt Morgan (1901) investigated the scaling of cellular structures in *Stentor* and found that after bisection, the cell is able to restore the proper scaling of its components in a matter of hours. This entails replacement of the oral apparatus in the anterior half with a new, smaller one, via the reorganization process. In Mob1 RNAi cells, if they are bisected early in the RNAi experiment, before the “medusoid” phenotype (see above) has become apparent, cell geometry rapidly becomes abnormal leading to an acceleration of the defect (Slabodnick et al., 2014). This observation suggests that regeneration after bisection places a particular burden on the Mob1-based signaling pathway beyond that required in normally growing cells, further implicating this pathway in maintaining and restoring proper cell organization.

One of the most striking visual features of *Stentor* is the orderly parallel striping of the body surface. By cutting into the cortex with glass needles and pushing pieces around, it is possible to rotate segments of the cortex out of alignment with the rest of the cell, or even to mince the whole surface into a patchwork quilt of striped sections, randomly aligned with each other (Tartar, 1960). Following these disarrangements, the *Stentor* cell is able to restore a normal pattern (Figure 5D), which it appears to do through a combination of stripe growth, stripe shrinkage, and annealing of stripes with matching widths (Tartar, 1956b). The key principle is the ability of parallel linear structures to elongate and then link up with other parallel linear structures. The microtubule bundles (Km fibers) in ciliates have been shown to undergo directional growth, elongating from their plus ends, and can even do so independently of the normally associated basal bodies in some ciliate species (Ng, 1979). Thus, one likely mechanism for restoring a parallel configuration of cortical rows would be for one or a few of the cortical domains to undergo growth by elongation of its rows, while other domains shrink, until eventually what is left is all aligned the same way. Such a mechanism would resemble the way magnetic domains grow and shrink when a material is magnetized, but it would potentially require a long-range interaction between neighboring domains such that growth would be favored among domains sharing a common orientation. A still open question is whether rotational motion of cortical fragments may also play a role in alignment. One can envision a process whereby a microtubule bundle from one fragment anneals to a bundle on another fragment, after which elastic forces would tend to drive the two fragments to rotate until their bundles are properly aligned.

## Compare and Contrast: *Stentor* vs. Animal Regeneration

Regeneration in *Stentor* takes place at an entirely different scale from regeneration in animal models. Because it is a single cell, there are no stem cell populations on which to draw, no neurons to transmit signals, no cells to migrate, and no cell-cell contacts to define regional identity. Everything has to be done within a single common cytoplasm. We thus imagine that *Stentor* regeneration must use entirely different mechanisms from classic models of animal regeneration. Whether animals may use *Stentor*-like mechanisms within their own cells to drive morphogenetic and regenerative processes at a cellular level is an entirely different question, that we will address in the Discussion section. Here, we point out a few examples of animal regeneration in which there are apparent similarities to regeneration in *Stentor*, albeit at a different scale.

Oral apparatus regeneration takes place at a defined location (the contrast zone) on the cell body, spatially separated from the OA. The OA is thought to generate an inhibitory signal that travels to the contrast zone and prevents a primordium from initiating regeneration when an OA is present (Figure 4). An analogous situation is seen in regeneration of eyestalks in crustaceans (Mykles, 2021). Many crustaceans can regenerate their eyestalks if they are severed, which one can imagine may happen rather frequently given the way the eyestalks project out from the head of the animal, unprotected by the thick carapace. This regeneration requires the animal to start molting its shell, which is regulated by a gland called the Y-organ, which secretes ecdysteroid hormones that regulate molting and regeneration. The secretory activity of the Y-organ is normally inhibited by peptide hormones produced in a neurosecretory gland called the X-organ. The Y organ is part of the brain, but the X-organ is located at the tip of the eyestalk. In an intact animal, the X-organ produces peptide hormones at the tip of the eyestalk which then travels to the brain, where it shuts off the Y-organ. This prevents eyestalk regeneration or molting. But if the eyestalk gets severed, then the X-organ is removed, and so there is no longer a source of the inhibitory hormones, so the Y-organ turns on and produces hormones that trigger regeneration and molting. The overall geometry of this situation clearly resembles the arrangement in *Stentor* where an inhibitory signal from the OA acts to prevent formation of an oral primordium at the stripe contrast zone subtended by the oral structures. In this model, the OA or some portion of it corresponds to the X-organ, which transmits a signal to the primordium corresponding to the Y-organ, with the ciliary rows anterior to the contrast zone forming a conduit for the signal much as the eyestalk serves to transmit the peptide signals in the crustacean case.

When *Stentor* regenerates a new oral apparatus, the oral primordium always forms in a defined location, the contrast zone, which evidently presents an appropriate molecular context to allow development of the oral primordium. In teleost fish, scales form within dermal spaces known as scale pockets (Meunier, 2002). When a scale is removed, it can re-grow, and this takes place only within existing scale pockets (Bereiter-Hahn and Zylberberg, 1993). The correlation between the location of scale pockets and the location of scale regeneration are not just coincidence: if scales are transplanted into empty pockets they can grow, but if they are transplanted elsewhere on the organism they erode. If half the scale pocket is cut away, the remaining pocket will form only half a scale (reviewed in Goss, 1969). Cells lining the scale pocket proliferate and condense to form the beginnings of a new scale (Iimura et al., 2012). The scale pocket thus serves as a defined location in which a scale can regenerate, a necessary signal to support scale formation and a source of material from which the new scale can be built. If the contrast zone is behaving similarly to the scale pocket, it raises an important unanswered question about OA regeneration in *Stentor*—is the contrast zone just a signal to tell the cell where to form the OA, or do the basal bodies present in the contrast zone serve as components from which to construct the new oral primordium?

Many instances of *Stentor* growth and regeneration rely on the fact that individual ciliary rows are self-propagating via the mechanism of centriole duplication. One example, discussed above, occurs when the cortex is disarranged by rotation or minceration, individual ciliary rows can grow and shrink so as to restore a normal parallel arrangement of stripes on the surface. The key underlying mechanism for restoring patterning to the cortex in such disarranged cells is the independent growth of parallel, polarized structures (the ciliary rows). A similar situation appears to hold in the regeneration of the fins of teleost fishes. These fins are composed of parallel rays made of cartilage. During normal fin growth, each fin ray elongates, and if one cuts through a fin, it can regenerate simply by elongating its fin rays, each of which continues growing (Akimenko et al., 2003). On the other hand, if one cuts the fin longitudinally, it can’t make new fin rays. In essence, the fin can regenerate because of each of its parallel longitudinal elements (the fin rays) can individually regenerate as an autonomous unit. The behavior of these fin rays is thus highly similar to the cortical ciliary rows of *Stentor*.

The cortex of *Stentor* and other ciliates is highly polarized along the A/P axis. I discussed above the tendency for the ciliary rows of the *Stentor* cortex to form parallel arrays with common polarity (plus ends at the anterior and minus ends toward the posterior of the cell) following minceration (Figure 5D). Looking at an even smaller scale (Figure 1B), the entire surface of the cell can be viewed as a lattice of cortical units, each consisting of a pair of basal bodies and joined by associated fibers to neighboring cortical units (Aufderheide et al., 1980). These cortical units are asymmetrical structures and in a normal cell they all have the same polarity, such that when the cilia beat, they beat in the same direction to drive forward motion of the swimming cell. This partitioning of the cell surface into a lattice of polarized units is highly reminiscent of planar cell polarity at the tissue scale (Eaton, 1997), in which a tissue is divided up into polarized cells, each of which has its PCP molecular pathway oriented in the same direction as its neighbors. It is known that the PCP pathway can respond both to extracellular fluid flow (Guirao et al., 2010) as well as to mechanical tension within a tissue (Aigouy et al., 2010). In *Stentor*, the cortical cilia generate a coherent flow over the whole body (Wan et al., 2020) which I hypothesize, based on the role of flow in PCP, might serve as a signal to help align the cortical rows during recovery after minceration or other disarrangements. It is interesting to note that *Stentor* regeneration is accompanied by expression of genes whose products are known to be involved in coupling ciliary orientation to planar cell polarity proteins in animals (Sood et al., 2021). Likewise, the *Stentor* cortex contains contractile fibers built of centrin-like EF hand proteins, and I hypothesize that mechanical tension generated by these fibers might play a role in transmitting long range spatial information to help enforce a common polarity among cortical units. In light of the discussion above, it is also interesting to note that PCP is involved in fish fin regeneration (Stoick-Cooper et al., 2007) as well as in many other regeneration paradigms such as in Planaria (Almuedo-Castillo et al., 2011). Given some of the phenomenological similarities between PCP and *Stentor* cortical polarization, mathematical modeling of PCP (Amonlirdviman et al., 2005; Burak and Shraiman, 2009) may serve as a basis for building models for *Stentor* surface patterning that can incorporate both short range interactions among cortical units and long-range interactions mediated by fluid flow or mechanical tension.

## General Issues of Regeneration Shared Between *Stentor* and Animals

A classic question in animal regeneration is whether a given regenerative process is a unique or special process, or simply a re-activation of normal developmental pathways. In *Stentor*, much of the existing evidence points to the latter possibility. For a cell, “development” can be viewed as equivalent to “cell division,” since that is when new structures must be developed such that both cells have all required structures. *Stentor* cells undergo a division process in which pre-existing cortical structures are retained while new structures are built (Tartar, 1961). The cell divides into anterior and posterior daughter cells, such that the anterior daughter inherits the OA and the posterior daughter inherits the holdfast. Prior to cytokinesis, a new OA is built in the posterior half of the cell, which then slots into the cytokinetic furrow to become the OA of the posterior daughter cell. This formation of an OA during division follows the same morphogenetic steps as seen in regeneration of the OA, suggesting the process may be the same. During cell division, the macronucleus changes shape from a long string of beads to a single compact blob. The reason for this shape change is not known, although it is speculated to mix the genomes of the highly polyploid nucleus to ensure equal partitioning during division. During OA regeneration, the macronucleus undergoes identical shape changes (Paulin and Brooks, 1975), again consistent with the idea that regeneration entails a re-activation of some developmental processes normally occurring in division. Finally, transcriptional analysis of genes expressed during regeneration all indicate the upregulation of mitosis-related genes (Sood et al., 2017; Onsbring et al., 2018; Wei et al., 2020). Taken together, it seems that, as in many examples of animal regeneration, regeneration in *Stentor* is actually telling us about developmental processes that are important even for cells not subject to damage.

Another classical question in the study of animal regeneration is whether a given structure is replaced by building new material (for example by trigger proliferation and differentiation of neoblasts) or by re-sculping existing material, for example via cell migration and trans-differentiation (Reddien and Sanchez-Alvarado, 2004). This question of morphallaxis vs. epimorphosis has not yet been answered in *Stentor*. Formation of the oral primordium clearly entails the appearance of thousands of basal bodies, but whether these form by new synthesis, or by re-purposing basal bodies from neighboring cortical ciliary rows, has not been determined. During formation of the oral primordium, there is a stage at which basal bodies constitute an “anarchic field” (Bernard and Bohatier, 1981), so-called because neighboring basal bodies appear to have lost the usual rotational alignment that they would normally have in cortical structures. This apparently random orientation of the basal bodies may suggest that they have recently formed by *de novo* assembly rather than by templated duplication, but it could also be consistent with a process in which pre-existing basal bodies in the ciliary rows break free from their normal positions and migrate to the anarchic field. There is precedent in other ciliates for pre-existing basal bodies to be re-tasked to build different structures, for example during cirrus duplication in *Paraurostyla* (Jerka-Dziadosz, 1980). In the case of the *Stentor* oral primordium, the loss of attachments of such re-tasked basal bodies to their neighbors would potentially result in random rotational orientations, explaining the anarchic field. Transcriptomic studies have found that during OA regeneration, genes involved in basal body biogenesis are upregulated (Sood et al., 2017; Onsbring et al., 2018; Wei et al., 2020). The simplest explanation for this observation would be that these genes are turned on in order to drive new basal body formation at the moment of regeneration, which would argue against a re-utilization model.

In animal development, we often distinguish between mosaic and regulative forms of development. Classically, these were distinguished in experiments in which early blastomeres were separated from each other and the subsequent fate followed. In strongly mosaic systems, the individual blastomeres have defined fates early on that cannot be changed, while in strongly regulative systems, it is possible for cell-cell interactions or other active pattern homeostasis mechanisms to restore a normal animal form starting from a sub-set of the blastomeres. That *Stentor* follows a regulative scheme is perhaps most clearly seen in cell fusion mass experiments, in which multiple cells are grafted together in random orientations. These fusion masses undergo dynamic rearrangements and eventually lead to a normal looking cell many times the size of a normal *Stentor* cell (Tartar, 1961).

We see that many of the regenerative processes in *Stentor* bear striking similarities to regenerative processes in animal models, and raise many of the same questions. We do not mean to suggest that the processes have the same molecular basis in both cases, nevertheless it is interesting to see how living systems deploy the same regulatory logic across vastly different scales of organization.

## Discussion

Most of the information about *Stentor* regeneration discussed above has come from microsurgical experiments, and our molecular understanding of the process remains very poor. Now that we have assembled the *Stentor* genome (Slabodnick et al., 2017) and developed methods for perturbing gene function by RNAi in *Stentor* (Slabodnick et al., 2014), the path is open to dissecting the molecular basis of regeneration and its regulation (Figure 5B). Current methodological challenges that still remain are developing methods for live cell imaging such large motile cells at high resolution, and the establishment of transgenics and genetic methods in Stentor. We can use RNA to knock down gene expression, but methods to express transgenes are still under development. Gene editing and genetics will require reliable methods for mating *Stentor* cells, something that remains problematic. Mating is well documented in this organism but it appears to happen spontaneously—conditions have not yet been developed to trigger mating. Establishment of clonal lines will be an important step toward developing genetics, since it will help to determine the number of different mating types. We are thus at a stage of the field where several key methods are already in hand, while others are still under development. But why should we study regeneration in *Stentor*? As mentioned in the introduction, regeneration of animals has played an important role in revealing mechanisms of normal development. Given how little we currently know about the origins of cellular geometry (Kirschner et al., 2000; Harold, 2005; Marshall, 2011), regeneration studies will have an important role to play for understanding single cell development.

To see how regeneration can influence our thinking about development, consider the possibility that cells grow like crystals, with new cellular structures templated directly by existing ones. Indeed, this has been demonstrated for the case of ciliary rows by Beisson and Sonneborn (1965), who found that inverted ciliary rows in Paramecium can be propagated indefinitely. This propagation takes place because the basal bodies of the ciliary rows dictate the position and orientation of new basal bodies, such that new basal bodies form immediately anterior to pre-existing ones (Dippell, 1968). The basal bodies are themselves inherently asymmetrical structures, which dictate the formation and orientation of associated fiber structures. Consequently, an inverted ciliary row grows by elongation while maintaining the inverted orientation of all the basal bodies, and associated structures, composing the row. When the cell divides, it does so transversely to the rows, such that each daughter cell inherits a new inverted row of half the length of the mother cell. Based on experiments like this, one could propose a model for cellular morphogenesis in which cells never actually form new structures, but rather inherit all of their organization from parent cells. In such a scenario, cells would never require mechanisms to break symmetry, establish polarity, or form new patterns—all the things we think of as representing “development.” Instead, they would simply grow and then partition existing patterning. But in such a scenario, regeneration would not be possible. Alteration in structure, or loss of a component, would result in a permanently inherited alteration or loss. The fact that *Stentor* can regenerate a normal cellular geometry after almost any perturbation thus strongly argues against a purely templated mechanism for maintaining cell geometry, and instead suggests that cells must constantly retain the ability to generate and correct patterning. Regeneration also provides a convenient way to trigger the need for these processes—the experimenter can force the cell to regenerate at a time of ones choosing, allowing the process to be quantified under various perturbations. The acute nature of surgical perturbation contrasts with the slower timescale of genetic perturbation, even when using conditional mutants. The fact that a surgical operation can be performed and the effects immediately observed reduces potential concerns about compensatory mutations. Thus, at the most general level, *Stentor* regeneration provides a way to study morphogenesis and patterning within a single cell that has a different set of advantages and disadvantages compared with traditional genetic model systems.

Among the lessons learned from *Stentor* regeneration is the fact that biological information resides not just in the genome, but also in the physical structure of the rest of the cell. The induction of new oral primordia by artificially constructed contrast zones (Figure 3) illustrates this point—simply by altering the physical relations between cortical regions, it is possible to create a cell with a radically altered structure (multiple oral apparatus) without any modification in the genome itself. Similarly, the ability to monitor the molecular pathways of regeneration following surgical perturbation of cell structure potentially opens a window into learning how a cell senses its own organization. For example, the fact that rotating the anterior half of the cell relative to the posterior half can trigger activation of the oral primordium strongly suggests a system that monitors the position of cortical structures relative to a longitudinal reference frame. But what sort of molecular mechanism can specify longitude in a cone-shaped cell? Whatever the mechanism, this may also provide the explanation for induction of new oral primordia in artificial contrast zones.

Will lessons learned in *Stentor* apply to other cell types? We have every reason to believe that they will. The *Stentor* genome contains remarkably few *Stentor*-specific genes (Slabodnick et al., 2017). On the contrary, the vast majority of *Stentor* genes have clear orthologs in other eukaryotes including animals. At a structural level, while linear ciliary rows are seen mostly in ciliates, the fundamental structural motif of a basal body or centriole pair which links to a set of associated fibers that emerge at defined angles relative to the centrioles, is highly conserved, being throughout eukaryotes including humans In humans, ciliated epithelia are organized by a lattice of molecular filaments in the cell cortex (see for example Kunimoto et al., 2012; Tateishi et al., 2017). The mechanisms that pattern such lattices are not fully understood, but there is clear conservation of molecular components between the human ciliated epithelial cell cortex and the cortical rows of *Stentor*. So at least in some specialized tissues, it is likely that some aspects of the molecular pathways of morphogenesis will turn out to be directly conserved. At a more conceptual level, many cells in the body face the same general challenge of the *Stentor* cell—how to establish complex, asymmetrical structures inside a single cell. Well known examples of complex cellular structures include the hair cells of the inner ear (Schwander et al., 2010) and the rod and cone cells of the retina (Kennedy and Malicki, 2009). The idea that *Stentor* and other ciliates are somehow unusual in their complex organization mainly arises from the fact that most commonly used cell culture lines take on an amorphous amoeboid appearance. But if we step back from the dish and look inside the body, complexity of cell structure abounds. Where does this structure come from? In some cases, organization in a cell may be induced by signals coming from neighboring cells, but in other cases it could easily arise cell-autonomously from developmental mechanisms that operate within the cells themselves, just as it does in *Stentor*.

By learning how *Stentor* cells regenerate their structure, it is hoped that new light may be shed on pathways for cellular morphogenesis that may also act within the many complex cells of humans. Many human diseases result from breakdown at the level of individual cells. Most work in regenerative medicine aims to replace damaged cells with new cells produced by differentiation of pluripotent stem cells. But this may be challenging in many cases because these new cells lack the context of the damaged cell to be replaced. An alternative strategy would be to learn how to encourage the damaged cells to repair themselves and regenerate their damaged structures. Identification of regeneration mechanisms and pathways in *Stentor* has the potential to suggest candidate pathways to explore for such a strategy in the context of human disease. At this point, such a suggestion remains highly speculative, however, and we close by arguing that the main motivation for studying *Stentor* regeneration is that it has been a long standing biological mystery for over a hundred years, and that breaking open such a mystery has clear potential for new fundamental insights into the origins of biological form.

## Author Contributions

The author confirms being the sole contributor of this work and has approved it for publication.

## Conflict of Interest

The author declares that the research was conducted in the absence of any commercial or financial relationships that could be construed as a potential conflict of interest.

## Publisher’s Note

All claims expressed in this article are solely those of the authors and do not necessarily represent those of their affiliated organizations, or those of the publisher, the editors and the reviewers. Any product that may be evaluated in this article, or claim that may be made by its manufacturer, is not guaranteed or endorsed by the publisher.
